# Biological exposure assessment to tetrachloroethylene for workers in the dry cleaning industry

**DOI:** 10.1186/1476-069X-7-12

**Published:** 2008-04-15

**Authors:** Lauralynn T McKernan, Avima M Ruder, Martin R Petersen, Misty J Hein, Christy L Forrester, Wayne T Sanderson, David L Ashley, Mary A Butler

**Affiliations:** 1 Centers for Disease Control and Prevention (CDC) National Institute for Occupational Safety and Health, 4676 Columbia Parkway, R-15, Cincinnati, OH 45226, USA; 2 University of Iowa Department of Occupational & Environmental Health, 100 Oakdale Campus, Iowa City, Iowa 52242, USA; 3 CDC National Center for Environmental Health, 4770 Buford Highway, F-47, Atlanta, GA 30341-3724, USA

## Abstract

**Background:**

The purpose of this study was to assess the feasibility of conducting biological tetrachloroethylene (perchloroethylene, PCE) exposure assessments of dry cleaning employees in conjunction with evaluation of possible PCE health effects.

**Methods:**

Eighteen women from four dry cleaning facilities in southwestern Ohio were monitored in a pilot study of workers with PCE exposure. Personal breathing zone samples were collected from each employee on two consecutive work days. Biological monitoring included a single measurement of PCE in blood and multiple measurements of pre- and post-shift PCE in exhaled breath and trichloroacetic acid (TCA) in urine.

**Results:**

Post-shift PCE in exhaled breath gradually increased throughout the work week. Statistically significant correlations were observed among the exposure indices. Decreases in PCE in exhaled breath and TCA in urine were observed after two days without exposure to PCE. A mixed-effects model identified statistically significant associations between PCE in exhaled breath and airborne PCE time weighted average (TWA) after adjusting for a random participant effect and fixed effects of time and body mass index.

**Conclusion:**

Although comprehensive, our sampling strategy was challenging to implement due to fluctuating work schedules and the number (pre- and post-shift on three consecutive days) and multiplicity (air, blood, exhaled breath, and urine) of samples collected. PCE in blood is the preferred biological index to monitor exposures, but may make recruitment difficult. PCE TWA sampling is an appropriate surrogate, although more field intensive. Repeated measures of exposure and mixed-effects modeling may be required for future studies due to high within-subject variability. Workers should be monitored over a long enough period of time to allow the use of a lag term.

## Background

Tetrachloroethylene (perchloroethylene, PCE) is widely used in industry as a dry-cleaning solvent, degreaser, and cleaner. PCE is a useful solvent in the dry cleaning industry because it is an effective cleaner and does not promote garment fading or shrinking. At least two-thirds of dry cleaners use PCE as a solvent in their dry cleaning operations [1]. Occupations in a typical dry cleaning facility include dry cleaning operators, spotters, garment pressers, counter workers, and delivery drivers. Operators load and unload the machines and receive the highest PCE exposure, while pressers who iron (press) and finish garments after washing have lower exposures.

The U.S. Occupational Safety and Health Administration (OSHA) regulates PCE exposure in air with an 8-hour time-weighted average (TWA) permissible exposure level (PEL) of 100 ppm (678 mg/m^3^) and a peak exposure limit of 300 ppm for no more than five minutes every three hours. The International Agency for Research on Cancer considers PCE a Group 2A probable human carcinogen [2] and work in the dry-cleaning industry as possibly carcinogenic to humans [3]. Documented health effects associated with PCE exposure range from central nervous system and reproductive effects to possible associations with several cancer sites [4,5] The American Conference of Governmental Industrial Hygienists (ACGIH) has recommended a threshold limit value (TLV) of 25 ppm over an 8-hour exposure and a biological exposure index (BEI) for PCE in end-exhaled air of 5 ppm [1]. Other recommended BEIs are 0.5 mg/L (500 ng/ml) PCE in blood and 3.5 mg/L trichloracetic acid (TCA), the major PCE metabolite, in urine. Exposure limits in other countries range from 5–100 ppm [2], with some countries setting exposure monitoring guidelines rather than exposure limits because of possible carcinogenicity. For example, the German Commission for the Investigation of Health Hazards of Chemical Compounds classifies PCE as a "substance for which in vitro or animal studies have yielded evidence of carcinogenic effects" with no acceptable exposure limit [6].

Approximately half of 244,000 dry-cleaning employees in the United States are women [7]. Because PCE is lipid-soluble and women typically have a higher proportion of body fat than men, it is anticipated that women would retain PCE longer than their male counterparts. This study was developed to focus on women's exposure to PCE as part of a pilot study investigating health effects as well as additional indices of effective biological exposure associated with PCE exposure in female dry-cleaning workers.

## Methods

The purpose of this study was to assess the feasibility of conducting biological PCE exposure assessment for employees in the dry cleaning industry. Because this investigation was a pilot study, sample size was limited, with a target of twenty exposed dry-cleaning workers and twenty referent laundry workers with no PCE exposure. Only dry cleaning facilities in southwestern Ohio were recruited for the study. Dry cleaning facilities were selected from an American Business Directory list of shops as of 1994 [8], and local telephone directory listings. Owners or managers of approximately 175 dry cleaning shops were contacted. Meetings were held with ten employers who consented to a visit to discuss the study in more detail and to conduct walk-through surveys. Facilities with more than five women employed on regular work schedules and with limited exposure to other chemicals (Stoddard solvent and spot-removing chemicals) were given priority.

After a facility was selected and the employer agreed to participation, recruitment meetings were held with female employees. Women who had worked at the dry cleaning facility full-time for at least one year were eligible to enroll in the study. Participants were compensated for their time and inconvenience at the end of the study.

A comprehensive sampling strategy was developed to assess both daily and cumulative PCE exposure through a variety of exposure indices. The overall sampling strategy included collecting multiple biological specimens and monitoring PCE at the workplace (Table 1). Repeated measures were collected to assess variability in PCE exposure on consecutive days (Wednesday, Thursday, and Friday) after at least two days of exposure. Additional specimens were collected from a subset of participants to assess PCE body burden before (Friday) and after (Monday) a non-working two-day weekend, for three sequential weeks (Table 2). Participants also provided information regarding demographics and work history. Body mass index (BMI) was calculated as weight (in kg)/[height (in m)]^2^, using measurements obtained at a physician's office during a health exam conducted as part of the pilot study.

**Table 1:** Participant core sampling schedule. Core sampling (n=18)

**Wednesday**	**Thursday**	**Friday**
Breath Sample AM	Breath Sample AM	Breath Sample AM
	Urine Specimen AM	
	Blood Specimen AM	
		
Personal Air Sample	Personal Air Sample	
		
Breath Sample PM	Breath Sample PM	
	Urine Specimen PM	

AM = morning specimen collected at home (urine) or just before entering the facility (breath, blood).

PM = afternoon specimens collected at the end of the workday inside (urine) and outside (breath) the facility.

**Table 2:** Participant weekend sampling schedule. Weekend sampling (n=13)

**Week**	**Monday**	**. . .**	**Friday**
1			Breath Sample PM
			Urine Specimen PM

2	Breath Sample AM		Breath Sample PM
	Urine Specimen AM		Urine Specimen PM

3	Breath Sample AM		Breath Sample PM
	Urine Specimen AM		Urine Specimen PM

4	Breath Sample AM		
	Urine Specimen AM		

AM = morning specimen collected at home (urine) or just before entering the facility (breath).

PM = afternoon specimens collected at the end of the workday inside (urine) and outside (breath) the facility.

Not all participants were sampled in four consecutive weeks

### Personal air sampling

Personal breathing zone samples were collected from each employee on two consecutive work days (Wednesday and Thursday) using NIOSH Method 1003 (precision [coefficient of variation] 5.2%, accuracy 15.1%) for halogenated hydrocarbons [9]. Each participant wore a personal pump with a 100 mg/50 mg coconut shell charcoal tube attached to clothing near her breathing zone. The personal breathing zone samples were collected throughout the workshift during 120-minute intervals with a flow rate of 0.1 liters/minute.

Charcoal tube samples were shipped to the analytical laboratory via overnight mail and were analyzed for PCE using carbon disulfide desorption with analysis by gas chromatography with flame ionization detection. The limit of detection ranged from 0.0008 to 0.002 mg/sample.

### Exhaled breath sampling

Alveolar breath sampling is a non-invasive procedure with a low probability of causing discomfort to the worker [10]. Alveolar breath samples were collected immediately before (prior to entering the facility) and following the workshift on Wednesday and Thursday and before the workshift on Friday. To test the feasibility of assessing PCE retention after stopping work, breath samples were also collected from a subset of participants at the end of each work week and before the beginning of the next work week, for three weeks.

Breath samples were collected according to NIOSH Method 3704 (precision 11.5%, accuracy 22.5%) [11]. Employees were asked to breathe normally for five minutes outside of any area containing measurable PCE in the air (i.e., outside the dry cleaning facility). They were then instructed to take a deep breath, hold it for 10 seconds, exhale half their breath (self determined) into the air and to then exhale the last half of their held breath into a 1 liter sample collection bag. This procedure was repeated until the collection bag was full.

Exhaled breath samples were transferred to a NIOSH laboratory for analysis. If the collection bags were cool, they were allowed to acclimate to room temperature (~23°C.). Each exhaled breath sample was injected into a gas chromatograph with a photoionization detector and analyzed for PCE. Samples could be stored without loss (<8%) for approximately eight hours. The limit of detection for the PCE in exhaled breath analysis was 0.009 ppm, and the limit of quantification was 0.031 ppm.

### Blood specimens

Following three consecutive days of PCE exposure, blood was drawn from participants in a temperature-controlled vehicle before they entered the facility for their work shift on Thursday. Following universal precautions, a trained phlebotomist drew 7 ml of blood from the antecubidal area (inside arm opposite the elbow) of the arm of each participant.

Aliquots were shipped in insulated containers and analyzed for PCE according to the standard protocol developed by the Air Toxicants Branch, Division of Laboratory Sciences, National Center for Environmental Health, CDC for the measurement of volatile organic compounds (VOCs) in human blood[12]. This purge and trap (direct sparging with helium) gas chromatographic method used high-resolution mass spectrometric detection in the full scan mode. The method is applicable to the determination of PCE in 10 mL blood at a detection limit of approximately 0.02 parts per billion (ppb). Quantification was achieved by isotope dilution in all cases by reference to commercially available labeled isotopes [12].

### Urine specimens

Urine specimens were collected after at least three working days of PCE exposure during the same week as the other biological specimens (Thursday and Friday). Each participant donated three urine specimens (samples pre- and post-shift on Thursday and post-shift on Friday, Table 1). To test the feasibility of assessing PCE retention after stopping work, a subset of participants also provided additional urine specimens: one on the last day of the work week, post-shift, and one on the first day of the following work week, pre-shift, for three consecutive weeks (Table 2).

Specimens were collected using the Commode Specimen Collection System^® ^(Virginia Design Packaging Corporation, Suffolk, VA) and transported to a NIOSH laboratory and shipped to a contract lab in insulated coolers on blue ice for TCA and creatinine analysis. For TCA analyses, urine specimens were treated with 0.5 M pH 8.5 phosphate buffer to hydrolyze TCA to chloroform. Headspace analysis was conducted using gas chromatography with electron capture detection to measure chloroform. Blank urine specimens spiked with TCA were used as external calibrations. The limit of detection for the TCA in urine analysis was 0.05 mg/L. Samples reported as 'less than the detection limit' were assigned a value one half the detection limit for analysis. Urine specimens with creatinine concentrations in the 0.3 – 3 g/L range were adjusted accordingly. In accordance with ACGIH guidelines, specimens with creatinine concentrations outside this range were excluded prior to analysis [1].

### Statistical methods

Statistical analyses used SAS Software (SAS Institute, Cary, NC). Because the blood and TWA outcomes were right-skewed, these variables were natural log transformed to obtain approximate normal distributions. No transformation was necessary for PCE in exhaled breath variables. (The distribution of breath PCE over the Wednesday – Friday data was slightly positively skewed. The distribution of the logs was moderately negatively skewed. i.e. taking logs made the situation worse. The residuals for the non-logged variable from some models were nearly symmetrical. Thus logs were not taken.)

TCA in urine variables were log transformed for correlations, but no transformation was needed or used for modeling. Geometric means and standard deviations were calculated for the measures of PCE in air, blood, exhaled breath, and urine. Pearson correlation coefficients were computed between the log of PCE TWA and various biomarker measurements. The PCE in exhaled breath measurements and TCA in urine measurements were examined to determine the effects of day of week and time of day (pre-shift or post-shift) and to adjust for subject. PCE in exhaled breath and TCA in urine measurements for three weekends were also used to evaluate the body burden after two consecutive days without PCE exposure (Friday and Monday concentrations).

Statistical models were used to relate same day post-shift, next day pre-shift, and next day post-shift PCE in exhaled breath to PCE TWA after adjusting for fixed effects of "day" and BMI. Since repeated measurements were collected over consecutive days, the MIXED procedure in SAS was used to account for the correlated nature of the data. The method of restricted maximum likelihood (REML) was used to estimate the covariance parameters, and the Kenward-Rogers option was used to compute degrees of freedom. The mixed-effects model was given by

Y_*ij *_= β_0 _+ β_1 _(Day = Thursday) + β_2_BMI_*i *_+ β_3 _ln(PCE TWA_*ij*_) + b_*i *_+ ε_*ij*_

where *Y*_*ij *_represents the concentration of PCE in exhaled breath for participant *i *at time *j*; β_0 _represents the intercept; β_1_, β_2 _and β_3 _represent the fixed effects of day, BMI, and natural log-transformed PCE TWA; b_*i *_represents the random effect for participant *i*; and ε_*ij *_represents the random error associated with participant *i *at time *j*. In the model, "day" indicates the day on which the PCE TWA measurement was collected. The models assumed that b_*i *_and ε_*ij *_were mutually independent and normally distributed with zero means and variances σ^2^_b _and σ^2^_w_, respectively, resulting in a compound symmetric covariance structure. The percent of variation explained by the fixed effects in the model was estimated by comparing the estimated total variance from the fitted model to the estimated total variance from the model that specified no fixed effects [13], and residual plots were used to confirm that models did not violate analytic assumptions.

### Informed consent

The entire study was reviewed and approved by the National Institute for Occupational Safety and Health (NIOSH) Human Subject Review Board. All participants provided written informed consent prior to inclusion in the study.

## Results

Eighteen female dry cleaning workers from four dry cleaning facilities participated. Three facilities used dry cleaning machines containing fourth generation technology with refrigerated condensers and carbon adsorbers; one used a machine with third generation technology containing only a refrigerated condenser [14]. Dry cleaning machine size varied from 30- to 60-pound drums, and machine age varied from nine to twelve years at these facilities. All women at a facility were sampled during the same calendar week; different facilities were sampled in different weeks. Demographic information for the participants is presented in Table 3. The mean age was approximately 41 years (range 22 – 68 years) and the mean BMI was 28 (range 21 – 37). Seventy-two percent of participants were white, and fifty-six percent were current smokers. The average tenure in the industry was approximately eight years (range 1 – 19 years). The majority of participants (n = 15) were pressers who finished garments by ironing or steaming and sometimes served customers at the counter during peak times. Only one participant was a full-time operator who loaded and unloaded garments from the dry cleaning machines while two other participants were part-time operators.

**Table 3:** Demographic characteristics of 18 female dry cleaning workers in the study

Characteristic	Mean ± Standard deviation	Range	Percent
Age (years)	41 ± 12	22 – 68	
Body mass index ^a^	28 ± 5	21 – 37	
Race (white)			72
Employment in industry (years)	8 ± 5	1 – 19	

^a ^BMI calculated as weight(kg)/[height (m)]^2^. Normal BMI for adult women is 19–25 kg/m^2^. BMI > 30 is considered obese [35]

Arithmetic and geometric means (M, GM) and standard deviations (SD, GSD) for each exposure index are presented in Table 4. Participants provided six exhaled breath samples during the sampling week: pre- and post-shift samples on Wednesday, Thursday, and Friday. In an analysis restricted to the 11 workers who provided measurements on all three days, post-shift PCE in exhaled breath increased gradually throughout the work week (GM (GSD) Wednesday: 0.94 ppm (1.58); Thursday: 1.38 ppm (1.90); Friday: 1.63 ppm (1.63)), but none of the differences were statistically significant. Participants provided three urine specimens during the week: pre- and post-shift samples on Thursday and a post-shift sample on Friday. For the urine specimens collected on Thursday, creatinine-adjusted TCA levels were not significantly different from pre-shift to post-shift.

**Table 4:** Summary statistics for PCE exposure indices for female dry cleaning workers^a^

PCE exposure indices	Wednesday			Thursday			Friday			Overall		
				
	n	M (SD)	GM (GSD)	n	M (SD)	GM (GSD)	n	M (SD)	GM (GSD)	n	M (SD)	GM (GSD)
				
PCE TWA (ppm)	17^b^	2.41 (3.42)	1.45 (2.73)	18	3.85 (5.35)	1.84 (3.84)				35	3.15 (4.51)	1.64 (3.26)
PCE in blood (ng/ml) pre-shift				15^c^	70.5 (106.4)	36.7 (3.34)				15	70.5 (106.4)	36.7 (3.34)
PCE in exhaled breath (ppm) pre-shift	15^b,d^	0.55 (0.38)	0.37 (2.87)	18	0.54 (0.41)	0.39 (2.40)	18	0.45 (0.33)	0.30 (2.82)	51	0.51 (0.37)	0.35 (2.65)
PCE in exhaled breath (ppm) post-shift	16^b,d^	0.87 (0.56)	0.71 (2.02)	18	1.14 (0.91)	0.71 (3.11)	11^e^	1.82 (0.93)	1.63 (1.63)	45	1.21 (0.87)	0.87 (2.51)
TCA in urine/creatinine (mg/g) pre-shift				14^e^	1.13 (1.94)	0.29 (7.23)				14	1.13 (1.94)	0.29 (7.23)
TCA in urine/creatinine (mg/g) post-shift				15^e^	0.86 (1.08)	0.37 (4.89)	9^e,f^	1.06 (0.93)	0.86 (1.86)	24	0.94 (1.01)	0.51 (3.89)

Abbreviations: n, number of samples/specimens; M, arithmetic mean; SD, standard deviation; GM, geometric mean; GSD, geometric standard deviation

^a ^For the referent laundry workers PCE TWA (nondetectable) and blood (two orders of magnitude lower than the dry cleaners) were reported in Toraason et al. 2003 [36]

^b ^One worker not available for sampling on Wednesday

^c ^Three blood specimen results did not satisfy laboratory quality control criteria and were excluded prior to analysis.

^d ^Exhaled breath samples were lost due to sample handling.

^e ^Samples contained creatinine concentrations outside the 0.3 – 3 g/L range and were excluded prior to analysis.

^f ^Two individuals worked alternate schedules and were not sampled on this day.

Pearson correlation coefficients are presented in Table 5. The concentration of PCE in the blood specimens obtained on Thursday was significantly correlated with PCE TWA from Wednesday and PCE in the post-shift exhaled breath samples from Wednesday. On both Wednesday and Thursday, the PCE TWA was significantly correlated with the same day post-shift, the next day pre-shift, and the next day post-shift PCE in exhaled breath samples. The Thursday PCE TWA was also significantly correlated with the Thursday post-shift creatinine-adjusted TCA concentration in urine. PCE concentration in post-shift exhaled breath samples was significantly correlated with same day post-shift creatinine-adjusted TCA concentration in urine on Thursday, but not on Friday.

**Table 5:** Pearson correlation coefficients for selected monitoring parameters^a ^in week 1

			Wednesday			Thursday						Friday		
					
			Preshift	Shift	Postshift	Preshift			Shift	Postshift		Preshift	Postshift	
					
			Breath	TWA	Breath	Breath	Urine	Blood	TWA	Breath	Urine	Breath	Breath	Urine
			
Wednesday	Preshift	Breath	1		0.58*	0.49	0.05	0.46		0.54*	0.74**	0.59*	-0.04	0.42
	Shift	TWA		1	0.81***	0.72**	0.33	0.84***	0.83***	0.66**	0.71**	0.59*	0.37	0.72*
	Postshift	Breath			1	0.79***	-0.07	0.88***		0.65**	0.59*	0.70**	0.31	0.72*
			
Thursday	Preshift	Breath				1	0.14	0.72**		0.86***	0.62*	0.90***	0.40	0.43
		Urine					1	0.37						
		Blood						1						
	Shift	TWA							1	0.72***	0.71**	0.54*	0.84**	0.36
	Postshift	Breath								1	0.61*	0.90***	0.65*	-0.17
		Urine									1			
			
Friday	Preshift	Breath										1	0.39	-0.10
	Postshift	Breath											1	0.20
		Urine												1

^a ^The following variables were log transformed: TWA (PCE TWA), Blood (PCE in blood), and Urine (creatinine-adjusted TCA in urine). Breath levels were compared throughout the test period. Urine metabolites were compared to previous or contemporaneous breath samples and TWAs. Blood levels were compared to previous or contemporaneous breath samples, urine specimens, and TWA.

* P-value < 0.05

** P-value < 0.01

*** P-value < 0.001

A subset of participants (n = 13) provided additional exhaled breath samples for three subsequent weekends: post-shift at the end of the work week and pre-shift prior to the start of the following work week. Geometric means and the median percent declines from Friday to Monday are presented in Table 6. Median declines from Friday to Monday ranged from 46% to 80%. After adjusting for weekend and participant, the overall decline in exhaled breath from Friday to Monday was statistically significant (P-value = 0.001). The same subset of participants also provided additional urine specimens for three subsequent weekends: post-shift at the end of the work week and pre-shift prior to the start of the next work week. The median declines in TCA in urine from Friday to Monday ranged from 3% to 38%. After adjustment for weekend and participant, the reduction in TCA in urine from Friday to Monday was not statistically significant (P-value = 0.12). There was no significant difference between those whose weekend measurements were done on three consecutive weeks and those with gaps of two weeks or more between weekend measurements (data not shown).

**Table 6:** Weekend change in PCE in exhaled breath and creatinine-adjusted TCA in urine

Parameter	Weekend	Post-shift Friday		Pre-shift Monday		Percent Change^a^	
				
		n	GM (Range)	n	GM (Range)	n	Median
			
PCE in exhaled breath (ppm)	1	12	1.48 (0.43 – 3.83)	13	0.66 (0.08 – 1.55)	12	-46%
	2	13	1.05 (0.22 – 4.42)	12	0.54 (0.17 – 0.97)	12	-53%
	3	13	1.83 (0.87 – 5.80)	11	0.34 (0.08 – 1.91)	11	-80%
	Overall	38	1.42 (0.22 – 5.80)	36	0.50 (0.08 – 1.91)	35	-58%
							
TCA in urine/creatinine (mg/g)	1	10	0.44 (0.03 – 2.24)	10	0.47 (0.05 – 6.24)	8	-25%
	2	11	0.72 (0.31 – 4.61)	11	0.51 (0.19 – 2.83)	10	-3%
	3	9	0.77 (0.31 – 1.64)	10	0.35 (0.02 – 0.90)	7	-38%
	Overall	30	0.62 (0.03 – 4.61)	31	0.44 (0.02 – 6.24)	25	-29%

Abbreviations: n, number of samples; GM, geometric mean

^a ^Percent change from post-shift Friday to pre-shift Monday is defined as 100% × (Monday – Friday)/Friday

Table 7 contains estimates of the fixed effects from the mixed-effects regression models relating same day post-shift, next day pre-shift, and next day post-shift PCE in exhaled breath to the natural log transformed PCE TWA. The relationship between PCE in exhaled breath and PCE TWA was significant in all three models. The day effect only reached significance in the next day pre-shift model. The percent of variance explained by the fixed effects in the model ranged from 47% for the next day pre-shift model to 63% for the next day post-shift model. In the final mixed models, the addition of the BMI term accounted for approximately 6%, 13%, and 5% of the variation in same day post-shift, next day pre-shift, and next day post-shift exhaled breath concentrations, respectively.

**Table 7:** Model summaries for regression models relating PCE in exhaled breath to PCE TWA

Model	Intercept	Day	Body mass index	ln (PCE TWA)	≈ R^2^
Same day post-shift (34 observations on 18 workers)	-0.46 ± 0.53 (0.40)^a^	-^b^	0.042 ± 0.018 (0.036)	0.52 ± 0.082 (<0.0001)	61%
Next day pre-shift (35 observations on 18 workers)	-0.42 ± 0.35 (0.25)	0.15 ± 0.051 (0.011)	0.028 ± 0.012 (0.038)	0.15 ± 0.045 (0.0032)	47%
Next day post-shift (28 observations on 18 workers)	-0.36 ± 0.72 (0.62)	-^b^	0.047 ± 0.025 (0.082)	0.66 ± 0.12 (<0.0001)	63%

R^2^, the percent of variance explained by the fixed effects in the model, is estimated by 1 – (estimated total variance for full model/estimated total variance for reduced model), where the reduced model contains only the intercept.

^a ^estimate ± standard error (P-value)

^b ^day effect was not significant and was excluded from the model

## Discussion

This study provided an opportunity to evaluate the logistics of conducting a biological exposure assessment in conjunction with a study of possible health effects for female workers employed in the U.S. dry cleaning industry.

Field assessments of occupational PCE exposure with biomonitoring have been conducted in dry cleaning [15-17] and other industries [18,19] and under experimental chamber conditions [20-22]. Table 8 presents biomonitoring results for our study and others of dry cleaners. Because protocols, facility equipment, and ambient exposure levels differed, and because most studies were small, across-study comparisons would not be meaningful. It should be noted that most of the biological measures reported for workers in facilities with dry-to-dry machines do not exceed BEI guidelines except for PCE in exhaled breath and blood in Gobba et al. [15].

**Table 8:** Summary statistics for PCE exposure indices in our study and other studies

PCE exposure indices	Present study (n = 18)		Gobba (n = 26) [15]		Solet (n = 195) [16]		Lauwerys (n = 26) [17]		Skender (n = 18) [37]	
					
	M (SD)	GM (GSD)	M (SD)	GM (GSD)	M (SD)	GM (GSD)	M (SD)	GM (GSD)	M (SD)	GM (GSD)
					
Machine type(s)	DD		DD		DD & T		Unknown		Unknown	
					
PCE TWA (ppm)	3.1 (4.5)	1.6 (3.3)	6.5 (6.4)		T29.5 (28.6) DD7.1 (6.4)	T 17.1 (3.6) DD6.5 (2.0)	20.8			
PCE in blood (ng/ml) pre-shift	70.5 (106.4)	36.7 (3.3)					400		0.6	
PCE in blood (ng/ml) post-shift			726 (937)				1200		1.5	
PCE in exhaled breath (ppm) pre-shift	0.5 (0.4)	0.4 (2.7)					1.9			
PCE in exhaled breath (ppm) mid-shift					T 7.7 (6.1) DD1.5 (1.2)	T 6.6 (2.3) DD2.2 (1.6)				
PCE in exhaled breath (ppm) post-shift	1.2 (0.9)	0.9 (2.5)	7.9 (12.1)				5.1			
TCA in urine/creatinine (mg/g) pre-shift	1.1 (1.9)	0.3 (7.2)							3.9	
TCA in urine/creatinine (mg/g) post-shift	0.9 (1.0)	0.5 (3.9)							3.4	

Abbreviations: n, number of samples/specimens; M, arithmetic mean; SD, standard deviation; GM, geometric mean; GSD, geometric standard deviation; DD, dry-to-dry; T, transfer

Some measurements from previous studies were converted from mg/L, mg/m^3^, mmol/mol, etc.

Previous studies have indicated that PCE in blood is the preferred biological index to monitor PCE exposures [17,18]. For investigators who want a quick, biological index to assess PCE burden, blood sampling is the optimum method; however, the invasive nature of blood collection may make recruitment more difficult. Since the majority of PCE exposure is inhalational, eight-hour TWA sampling is an appropriate surrogate for absorbed dose, absent data on individual metabolic factors. Indeed, a strong correlation was observed between PCE TWA and PCE in blood. Personal sampling avoids the complications of biological sampling, but requires more time for sample collection. For researchers wanting a less invasive biological index, the same day post-shift exhaled breath sample is an appropriate option [15]. Both the Pearson correlation and the mixed model results indicated that the same day post-shift exhaled breath was significantly related to the PCE TWA. A disadvantage of the exhaled breath sampling method used in this study was that sample analysis was rather labor-intensive. Other researchers have utilized an alternate exhaled breath technique with success [23]. We do not recommend using spot urine specimens to estimate PCE body burden, as we observed high variability in TCA urine levels.

PCE in post-shift exhaled breath generally increased throughout the week, although differences were not statistically significant. PCE body burden, as assessed by both urine specimens and exhaled breath levels, decreased appreciably after two days away from the workplace; however, the decline in creatinine-adjusted TCA in urine concentration was not statistically significant. The variability of TCA in urine indicated that 24-hour urine specimens might be more useful than the spot urine specimens employed in this study. Participants exhaled a residual amount of PCE in their breath before the start of the new work week. Given the levels of BMI observed in this study and the fact that PCE is lipid soluble, it is not surprising that PCE was retained in the body after a short hiatus in exposure.

The mixed model analysis provided additional insight into PCE exposures and pathways in this study. The primary advantage of the mixed model was that it allowed an analysis of repeated measurements from individuals and took into account the potential dependencies among the repeated measures. The mixed model also allowed use of all of the data and did not exclude individuals who did not provide all of the repeated measures. Others have shown the value of mixed models for analyzing repeated measures data in the presence of missing values [24-26] Exposure variability is important when assessing the value of a particular exposure measure [27]. The between-worker variation may reflect metabolic differences among workers that result in varying biological dose.

An important caveat of the mixed models utilized is that the measurements were collected on two consecutive days after at least two days of exposure. Given that the half-life of PCE in exhaled breath is known to be longer than 24 hours, the previous days' exposure may have affected the exhaled breath concentrations, but could not be taken into account by the model. The PCE TWAs from Wednesday and Thursday were highly correlated, as were the exhaled breath measurements. Although the model assumed a compound symmetric error structure, two repeated measurements are not sufficient to include a true lag term in the model. The Pearson correlation coefficients between PCE TWA and next-day post-shift exhaled breath were also significant (Table 5). This relationship may indicate that ambient exposure conditions and exhaled breath measurements from one day to the next are highly related to one another. Despite these potential limitations, the mixed models were still useful because they combined two days of exposure and illustrated the significant relationship between the PCE TWA and exhaled breath measurements.

Older studies had reported geometric mean PCE TWA values of 3.3 and 10 ppm for pressers and 13 ppm in dry cleaning operations [16,28] More recent studies (of facilities with more modern equipment) have reported concentrations near third- and fourth-generation machines (such as our participating shops used) ranging from 10–100 ppm (mean 40) and 4–54 ppm (mean 20), respectively [29]; PCE TWA values of 0.5–1 ppm for pressers and 4–5 ppm for operators [30]; and a majority of workers exposed at <5 ppm [31]. The geometric means observed in this study were similar to these recently published TWA values. At these ambient levels, elevated PCE concentrations were still evident in blood and breath and exhibited a strong correlation with PCE TWA.

### Study limitations

Facilities as well as participants were self-selected. That is, some facilities, with possibly higher PCE levels than those we observed, declined. If a facility declined we could not recruit workers from that facility. (In their study of 243 unionized dry cleaners in Michigan, Solet and Robins were able, with union assistance, to recruit 39 workers from nonparticipating plants [32]; none of the facilities we solicited were unionized.) Although the low concentrations observed may typify the reduction in PCE exposure due to improved technology, they may not be reflective of the industry as a whole. Participating shops had third- and fourth-generation machines while a 1995 survey found only one-third of U.S. dry cleaners had machines this advanced [33]. In addition, machine operators, who have higher exposure than other employees, [28,30] are more often male. Since this study focused on female employees we did not solicit participation from male machine operators.

Inability to schedule monitoring for all participants in a facility at once was a logistics obstacle. Conducting a study in a number of small retail establishments with a few workers each differed markedly from conducting a study in a large factory. Where there were only a few workers, days and hours of work tended to vary from worker to worker and from week to week. It was difficult to enroll a large number of employees who worked the same three consecutive days, causing data collection to extend longer than anticipated. Inclement weather adversely affected the volume of clothing processed, and if clothing volume was low, work hours were reduced accordingly. This may partially explain why exposures observed in this study were lower than those in other published studies, as previously mentioned. Blood collection was done on Thursday as we had observed, in other studies of dry cleaners and in work done in preparation for this study, that attendance on Fridays was spotty and those working on Friday usually left early. For these reasons we did not strictly follow the then-current ACGIH recommendations to collect blood specimens and end-exhaled breath prior to the last shift of the workweek [34].

Collecting pre- and post-weekend samples and specimens was drawn out by constantly changing participant work schedules (weeks, days, and hours worked). One facility with five workers was not included in the pre- and post-weekend sampling because of distance from our laboratories; however, collecting samples even from nearby facilities was complicated. This difficulty would need to be carefully evaluated when designing future efforts. Given the comprehensive nature of the sampling strategy and small numbers of employees at each facility, significant resources were devoted to sample collection. A more streamlined approach would be more efficient and mandatory for a larger study.

Prior to sample collection, work practices were observed during a brief industrial hygiene walkthrough. During personal sampling, several work practice changes were made to reduce worker exposure. The act of observing and sampling encouraged closer adherence to good work practices than usual. Although it is positive to have lower exposures, we question whether our values represent 'typical' conditions.

## Conclusion

This study provided a comprehensive PCE exposure assessment for a small sample of women working in the dry cleaning industry. This exposure assessment provides valuable insight for the development of a larger, more comprehensive study of the dry cleaning industry. If recruitment is not difficult, PCE in blood is the preferred biological index to monitor exposures. PCE TWA sampling is an appropriate surrogate, although more field intensive. Repeated measures of exposure and mixed-effects modeling may be required due to high within-subject variability. Workers should be monitored over a long enough period of time to allow the use of a lag term.

## Abbreviations

American Conference of Governmental Industrial Hygienists, ACGIH; biological exposure index, BEI; body mass index, BMI; geometric mean, GM; geometric standard deviation, GSD; arithmetic mean, M; U.S. National Center for Environmental Health, NCEH; U.S. National Institute for Occupational Safety and Health, NIOSH; U.S. Occupational Safety and Health Administration, OSHA; tetrachloroethylene (perchloroethylene), PCE; permissible exposure limit, PEL; parts per million [parts of air], ppm; estimated standard deviation, SD; trichloroacetic acid, TCA; threshold limit value, TLV; time-weighted average, TWA; volatile organic compound, VOC.

## Competing interests

The author(s) declare that they have no competing interests.

## Authors' contributions

LTM oversaw specimen collection, conducted breath analyses, contributed to the original protocol and wrote the paper. AMR contributed to the original protocol, participated in the field study, and revised the paper. CLF, WTS, and MAB contributed to the original protocol, participated in the field study, and reviewed the paper. DLA contributed to the original protocol, oversaw analyses of PCE serum levels, and reviewed the paper. MRP conducted many of the statistical analyses, reviewed the paper, and contributed to the revised paper. MJH conducted the mixed-effects model and other statistical analyses and reviewed the paper. All authors read and approved the final manuscript.
